# Nanobubble size distribution measurement by interactive force apparatus under an electric field

**DOI:** 10.1038/s41598-023-30811-9

**Published:** 2023-03-04

**Authors:** Zhenyao Han, Hao Chen, Chunlin He, Gjergj Dodbiba, Akira Otsuki, Yuezhou Wei, Toyohisa Fujita

**Affiliations:** 1 School of Chemistry and Chemical Engineering and College of Resources, Environment and Materials, Guangxi University, Nanning, 530004 China; 2 Graduate School of Engineering, The University of Tokyo, Bunkyo, Tokyo, 113-8656 Japan; 3 Facultad de Ingeniería y Ciencias, Universidad Adolfo Ibáñez, Diagonal Las Torres 2640, 11 Peñalolén, 7941169 Santiago, Chile; 4 Waste Science and Technology, Luleå University of Technology, 971 87 Luleå, Sweden; 5 School of Nuclear Science and Technology, University of South China, Hengyang, 421001 Hunan China

**Keywords:** Biochemistry, Environmental sciences, Solid Earth sciences, Chemistry, Engineering, Materials science, Nanoscience and technology, Physics

## Abstract

Nanobubbles have been applied in many fields, such as environmental cleaning, material production, agriculture, and medicine. However, the measured nanobubble sizes differed among the measurement methods, such as dynamic light scattering, particle trajectory, and resonance mass methods. Additionally, the measurement methods were limited with respect to the bubble concentration, refractive index of liquid, and liquid color. Here, a novel interactive force measurement method for bulk nanobubble size measurement was developed by measuring the force between two electrodes filled with bulk nanobubble-containing liquid under an electric field when the electrode distance was changed in the nm scale with piezoelectric equipment. The nanobubble size was measured with a bubble gas diameter and also an effective water thin film layer covered with a gas bubble that was estimated to be approximately 10 nm based on the difference between the median diameter of the particle trajectory method and this method. This method could also be applied to the solid particle size distribution measurement in a solution.

## Introduction

Nanobubbles are well-known as ultrafine bubbles finer than 1 µm^1^. Due to their small size, the electronic and lattice system to produce nanobubble^2^, and the large specific surface area, ultrafine bubbles have various special properties that completely differ from the original bulk materials in optical^3^, electromagnetic^4^, acoustic^5^, thermal^6^, and other physical properties^7–11^. Micro/nanobubbles are widely applied in environmental purification^12^, cleaning^13^, medicine^14^, agriculture^15^, aquaculture^16^, the food industry^17^, etc. Their performances strongly correlate with their size distribution; therefore, accurate measurement of the size distribution of nanobubbles is important. However, with the particle size distribution measuring equipment, the estimated size is different from the measurement methods. Additionally, it is necessary to measure not only the wide size range from nm for small bubbles to µm for large bubbles but also the bubble size in the condition of a very dilute concentration of bubbles to a high concentration of bubbles. In addition, it is important to measure the bubble size considering not only the similar reflection index of gas and liquid but also the liquid that does not pass laser light. Therefore, a simple automatic bubble size measurement method is needed. Here, to improve the above-mentioned problems, a novel, low-cost and simple method to measure the size distribution of micro/nanobubbles/particles, referred to as the interactive force apparatus (IFA), has been developed^18–23^. In this study, for the first time, the physical mechanical nanobubble size distributions were measured.

In the literature, the following nano-to-microbubble size apparatus has been mainly employed: high-speed camera image analysis^24,25^, electrical signal^26^ (Coulter counter), resonance mass^27,28^, particle trajectory (Nanoparticle Tracking Analysis (NTA)^29,30^, laser diffraction^31,32^, and dynamic light scattering (DLS)^33,34^ methods. The measurement size range is limited in each method (refer to Supplementary Fig. S1). To measure particles less than 100 nm in size, DLS, particle trajectory, laser diffraction, and resonance mass were selected. DLS and NTA have often been employed to measure nanobubbles^31,32,35–37^. In this study, the bubble size distributions were measured from low to high concentration and small nm to large µm. The specific generators to prepare nanobubbles at optimum pH and generation time were used to obtain the same concentration and size of nanobubbles. The IFA data were compared with the DLS and NTA data, which are the most prevalent data, to confirm the reliability of the IFA measurement data.

To produce nanobubbles, the following method was utilized. Hydrodynamic cavitation uses high-speed mixing^37^ and venturi-type^38^, gas oversaturation using solvent exchange and temperature change^39^, depressurizing-type^1^, membranes using small holes^40^, ultrasonic generators^41^, electrolysis^42^, etc. In this study, high-speed mixing, depressurizing time, membranes, and ultrasonic methods were utilized to produce nanobubbles.

In the IFA, the nanobubble size distribution was obtained by measuring the force between two electrode plates under a direct current (DC) electric field with an nm distance change (the details will be shown in the force measurement section). In atomic force microscopy (AFM)^43^, a single particle/bubble system is measured^44,45^. The measurement method in this paper can measure a more complex bubble/particle system by changing the electrode plate distance. However, many kinds of bubbles/particles can be measured by changing the electrode plate distance. In previous studies, we measured different types of mainly solid particles by IFA^18–23^ (refer to Supplementary Table S1). The mean particle size range was from 3 nm for the emulsion to 5000 nm for solid particles (as shown in Fig. S1 to compare the measurement size range). However, for IFA measurements, various kinds of bubble measurements, particle size simulations based on theory (as shown in Fig. S2), and comparisons with other methods, such as NTA, have not been described. In this study, the nanobubble size distribution measurement principles and the automatic nanobubble size measurement method with IFA are described. The effects of nanobubble size compared with the preparation method, oleic ion surfactant addition in water, and kerosene as the representative of non-polar organic liquid that shows the layer surrounding bubbles composed of hydrogen carbon are discussed considering the stability of the layer surrounding bubbles.

On the other hand, nanobubbles are known to show long-life stability^46^ in the condition of large zeta potential on the surface of nanobubbles. To prove the stability of nanobubbles, various concepts of the layer surrounding the gas nanobubble have been reported in the literature (Supplementary Table S2). For example, electrical double layers, hydrogen bonds, contaminants, and organic skin or films with surfactants have been reported. In this study, the hydrodynamic diameter measured by IFA and boundary layer thickness determined with NTA were measured for the first time. On the other hand, with the help of DLS and solvent relaxation NMR measurement results, the boundary layer thickness of nanobubbles is calculated to be about 40 nm^47^. In the large microbubbles, the critical film thickness by bubble rupture was reported to be approximately 30 nm in 10 µm sodium dodecyl sulfate (SDS) and 0.3 M NaCl solution^48^. However, the measurement results of the boundary layer thickness around the bubble by IFA differ from the above-mentioned reference^47,48^.

## Methods and materials

### Equipment preparation

The electric balance was a µg valance BA-T by A&D Company, Limited, Japan. The piezoelectric rod was a laminated piezoelectric actuator element fabricated by TOKIN, Japan. The controlled stage under the piezoelectric rod was LZ60 by Huike, China. The controller is FINE-503, SIGMA KOKI CO., LTD, Japan.

### Apparatus

**Figure 1:**
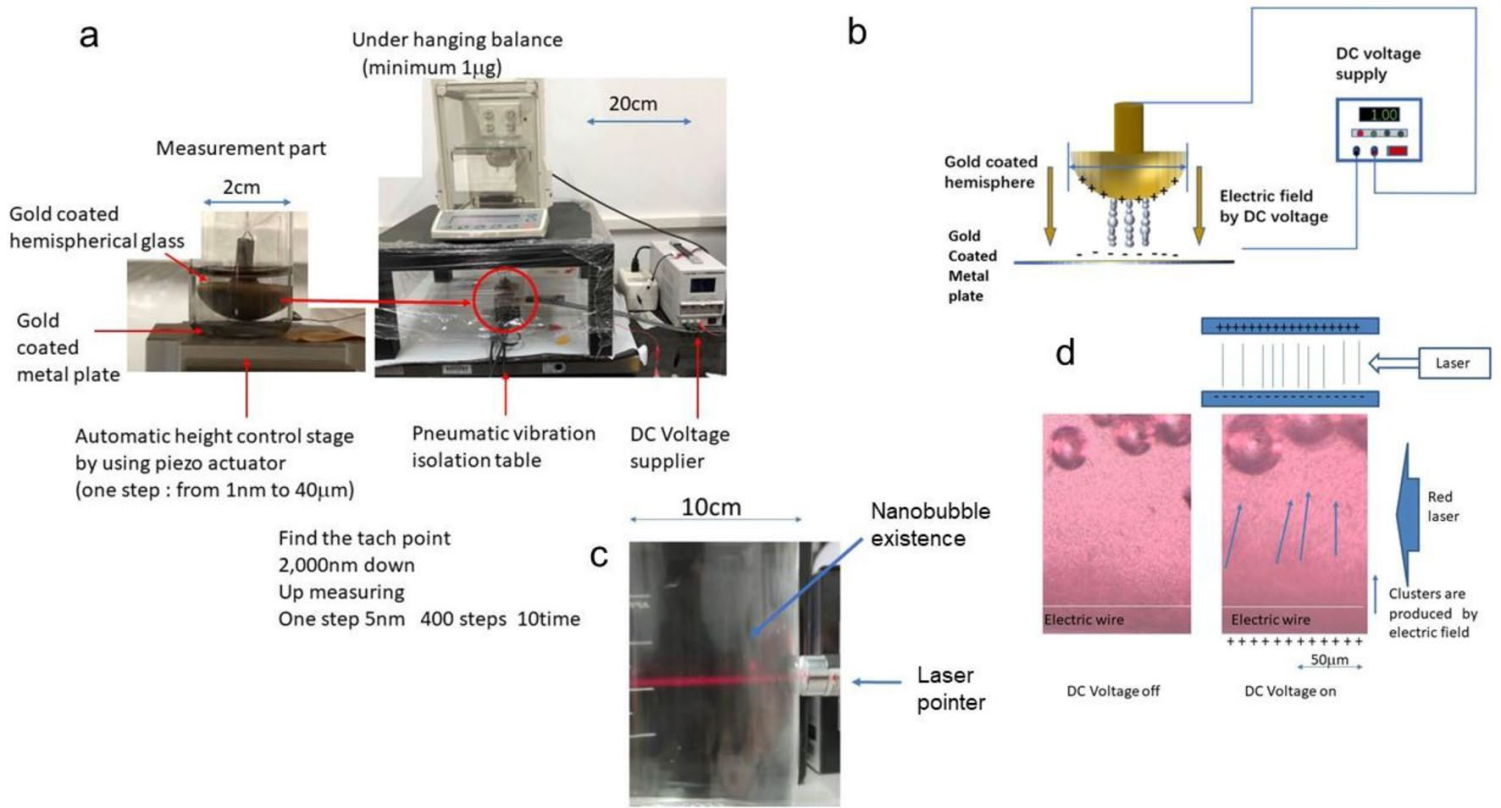
Experimental apparatus. (**a**) Photograph of the IFA apparatus used to measure nanobubbles. The left photograph shows the gold-coated flat plate and hemispherical glass plate in a nanobubble-containing fluid. The diameter of the hemispherical glass plate is 2 cm. The force between the hemispherical glass plate and the flat plate was measured by applying an electric field under a hanging electric balance (minimum 1 mg). (**b**) DC voltage supplier (right photo) applied an electric field between the flat plate and the hemispherical plate. The distance between the flat plate and the hemispherical plate is controlled from each 1–40 nm step using a piezoelectric stage fixed under the flat plate. The nanobubble shape is considered spherical both in the absence and presence of dielectric polarization. (**c**) The red laser pointer shows a distinct line indicating the existence of nanobubbles due to the Tyndall effect. (**d**) Existing nanobubbles formed clusters in the direction of the electric field under the application of a red laser (635–690 nm) (Here, the electric field is applied to the containing nanobubbles liquid set between two cylindrical wires).

The measurement method is shown in Fig. 1. The liquid-dispersing nanobubbles or solid particles selected to measure the size distribution are put into a cylindrical cell with a gold-coated, flat, brass plate at the bottom and then placed between a conductive, gold-coated, hemispherical glass plate and the flat plate^49^. The gold-coated hemispherical glass plate is hung under an electric balance, and the measured weight is stored in a computer as a voltage signal (Fig. 1a). Once a DC electric field is applied, the nanobubbles are arranged in the direction of the electric field due to their dielectric polarization (Fig. 1b). The electric field is not homogeneous. However, the largest electric field is applied between the closest electrodes. The piezoelectric stage under the sample cell can change the distance between the hemispherical plate and the flat electrode plate in the optimum periodical distance (the step length of each movement of the piezoelectric stage), such as several nm, and the electric balance weight changes due to the changes in the interaction between the hemispherical plate and the flat plate; now, we refer to them as electrodes. The electrodes were not touched at the starting point, but their closest distance was detected by measuring and identifying the significant resistance reduction.

The distance between the electrodes, obtained by changing the piezoelectric stage by controlling voltage, is stored in the computer as a voltage signal. Therefore, the laser beam could scatter the air nanobubbles due to the Tyndall effect and show the line in the water (Fig. 1c). In the microscope observation, the scattered small bubbles formed clusters in the direction of the electric field, and the large bubbles moved to the high electric field region by the electric field gradient. (Fig. 1d).

### Nanobubble preparation

Four types of preparation methods were used to prepare nanobubbles. The first generator is High-speed cavitation equipment (homemade equipment) by the spiral flow. The second one is an ultra-nanobubble generator by pressure difference. The third one is an ultrafine bubble by porous media (tube). The fourth one is an ultrasonic generator. The photographs of the preparation apparatus and methods are shown in Fig. S2 (Supplementary Note 4).

The above-mentioned nanobubble was prepared in deionized water and controlled the pH with sodium hydroxide aqueous solution. Next, the nanobubbles in sodium oleate solution were prepared by high-speed cavitation equipment (Fig. S2a) after controlling at pH 10. Finally, the nanobubble in kerosene was prepared by porous media (tube) by blowing the gas directly (Fig. S2c).

### Selected solid materials

Gold nanoparticle (~ 100 nm) standard solution (Zhengzhou Feynman Biotechnology Tech Co., Ltd.) was used to prepare a gold nanoparticle dispersion system for particle size measurement. Sodium oleate as a surfactant was obtained from Sinopharm Chemical Agent Co., Ltd. The regent grade of kerosene was purchased from Guangdong Guanghua Technology Co., Ltd.

### Measured equipment

Measurement of particle size by dynamic light scattering and zeta potential measured via the microelectrophoresis method was performed using a DLS system (NanoBook Omni, Brookhaven Instruments, Holtsville, NY, USA). In addition, the particle size was measured by the trajectory method, and the nanobubble number density (NTA) was obtained from NanoSight, NS300, Malvern (Worcestershire, UK).

## Results and discussion

### Concept and simulation of force measurements

**Figure 2:**
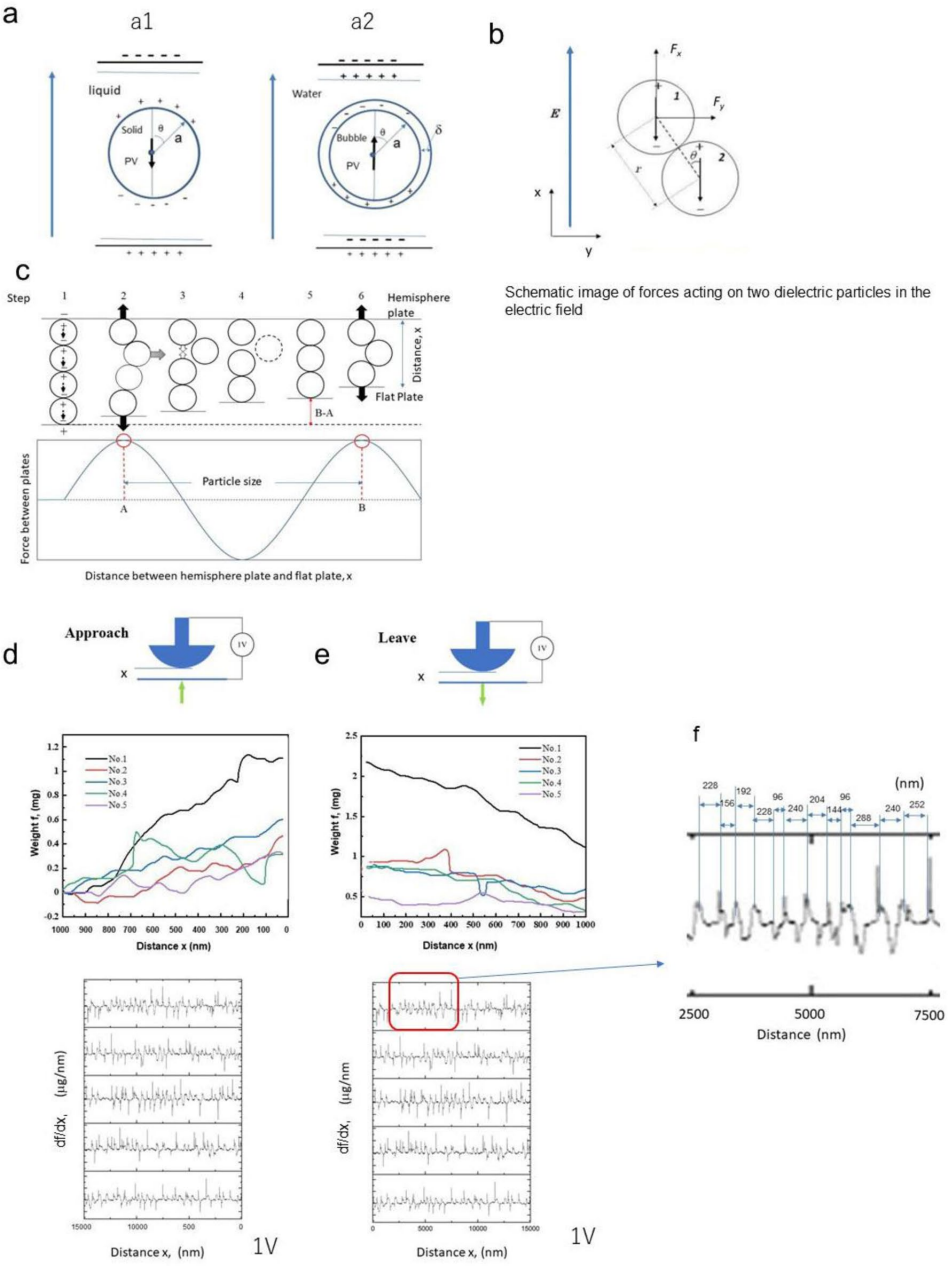
Particle interaction and movement and measured force. (**a**) (**a1**) shows a model of a solid particle in a fluid under an electric field, and (**a2**) shows a model of a nanobubble in water under an electric field. *δ* is the cover layer of the bubble. There are some explanations as the covered layer was discussed using Table S2. Here, the electrical double-layer and hydrogen bonds would be mainly affected. (**b**) Contacted two spherical particles with an induced electric dipole moment in the centre of the particle under an electric field. (**c**) The model shows the changing positions of four to three spherical particles by decreasing the distance between two electrodes. The model below shows the force change between two electrode plates by changing the particle positions. (**d**) The top figure shows two electrode plates sandwiching nanobubbles containing fluid approach. The weight results measured by the balance were converted to the force value using Eq. (2). The second figure shows the weight as the interactive force “*f*” of the electric balance shown in Fig. 1a, depending on the decreasing distance *x* under 1 voltage. More than 5 measurements were taken. The third figure shows “d*f*/d*x*” depending on x. (**e**) The top figure shows that two electrode plates sandwiching nanobubbles containing fluid leave. The second figure shows the weight “*f*” of the electric balance shown in Fig. 1a depending on the increasing distance x under 1 voltage. Five measurements were taken. The third figure shows “d*f*/d*x*” depending on *x*. (**f**) Enlarged view of “d*f*/d*x*”. The peak-to-peak distance shows the particle diameter.

The behavior/interactions among spherical dielectric particles/bubbles under an electric field are shown in Fig. 2a–c. The solid dielectric particle is charged in the electric field direction, and the dipole is shown in the center of the particle. For example, in Fig. 2a, the spherical dielectric particle under an external electric field *E* is estimated to have an induced dipole moment *m* in the center of the particle^50^ in the following formula:

1 $$m = PV$$

where* P* is the polarization of the particle and *V* is the particle volume. When the water surrounding a bubble is charged by applying an electric field, the dipole moment is considered to exist in the center of the spherical bubble.

In Fig. 2a1, the solid particle radius *a* does not include the liquid layer. The surrounding electric double layer on a solid particle is not considered in IFA. However, the measured radius of a nanobubble in IFA is the combination of the radius of a gas particle radius *a* plus the thickness of the layer around the nanobubble *δ* (Supplementary Table S2), as shown in Fig. 2a2. The forces between two spherical dielectric particles/bubbles in the direction of the electric field, as shown in Fig. 2b, are shown in *F*_*x*_ and *F*_*y*_ by the interaction of two particles; the equation depends on the particle position *x* and* y* (refer to Supplementary Note 5). The measured force was contributed by force *Fx*. For example, as shown in Fig. 2c, when 4 spherical particles/bubbles are arranged in the direction of the electric field, the dipole moment interaction under an electric field appears. The repulsive force dominates until a particle/bubble is released (steps 1 to 2 in Fig. 2c). Next, the electrode is attracted to the charged bubble until 3 particles are touched between the plates (steps 3 to 5 in Fig. 2c). Then, the repulsive force between the electrodes again appears between the electrodes (step 6 in Fig. 2c). The repulsive force appeared by decreasing electrode distance and when the particle was attracted between the space of two dipole particles. The peak-to-peak distance of forces corresponds to the particle/bubble diameter in IFA measurement. Though there are many clusters between two electrodes, the nearest distance nanobubble interaction force between electrodes is the largest and contributes to the force measurements. When the force is differentiated, the peak-to-peak corresponds to the particle/bubble diameter, as the differentiation shows a clear change of attractive and repulsive forces.

The simulation of force and size distribution measurement by IFA was also performed to validate this method. Here, the force between electrodes was confirmed by calculating the interaction between particles having dipole moments. When many different sizes of spherical particles were arranged between two electrodes, the particle force as a function of the electrode distance was calculated (refer to Supplementary Note 6). The particle size distribution could be obtained from the simulation by using the concept of Fig. 2. The forces between bubbles changing several parameters, such as particle size and distribution, applied voltage, and surrounding liquid permeability.

Here, the forces between two particles depended on the smallest distance between the hemispherical plate and the flat plate in the direction of the electric field.

### Force measurement

Using the apparatus shown in Fig. 1a, the forces between two electrodes were measured when the electrode approached and left. The lower electrode is controlled by the console and moves upward, while the upper electrode is not moved. The results are shown in Fig. 2d–f. If there are no nanobubbles in distilled water, a clear vibration did not appear. The total number of statistical data is more than 300, which is relatively conclusive.

The measured interactive force “*f*” is converted to the interactive force using the Derjaguin approximation^51^ of the following equation.

2 $$f {/} R = 2\pi W$$

where *R* is the curvature radius of the hemispherical glass and *W* is the interactive free energy. The interactive force *f* was increased as the electrode distance decreased. d*f*/d*x* is expressed as many peak-to-peak waves, as shown in one particle diameter.

The optimum electric field to measure the interactive force was examined by applying a voltage from 0.2 to 1 V at a distance greater than 800 nm. However, when the starting electrode distance was smaller than 800 nm, the measurement result was inaccurate because only small bubbles could be measured. Therefore, the voltage was compared to determine a more suitable measurement.

As a result, clear weight changes depending on the distance that appears at each voltage. The median diameter (50% of the particle diameter in the cumulative size distribution) was almost uniform at approximately 150 nm from 0.2 to 1 V (refer to Supplementary Note 7 for the voltage effect of the nanobubble size distribution by IFA). Our experimental measurement found that the 0.2–1 V had little influence on the measurement results, especially since the 1v measurement results were closer to the NTA results.

### Particle/bubble size measurement by IFA

The less polydispersed gold particles were measured to confirm the solid particle measurement by IFA instead of nanobubbles, as shown in Fig. S5 (Refer to Supplementary Note 8 for solid-gold, nanoparticle size distributions comparing DLS, NTA, IFA, and SEM). The SEM photo showed that the dispersed gold particles in water were approximately 100 nm. The median diameter of the obtained cumulative size distributions of DLS, NTA, and IFA were similar at 90 nm. The measured size distribution results by these three measurement methods were consistent with that measured by the SEM images.

**Figure 3:**
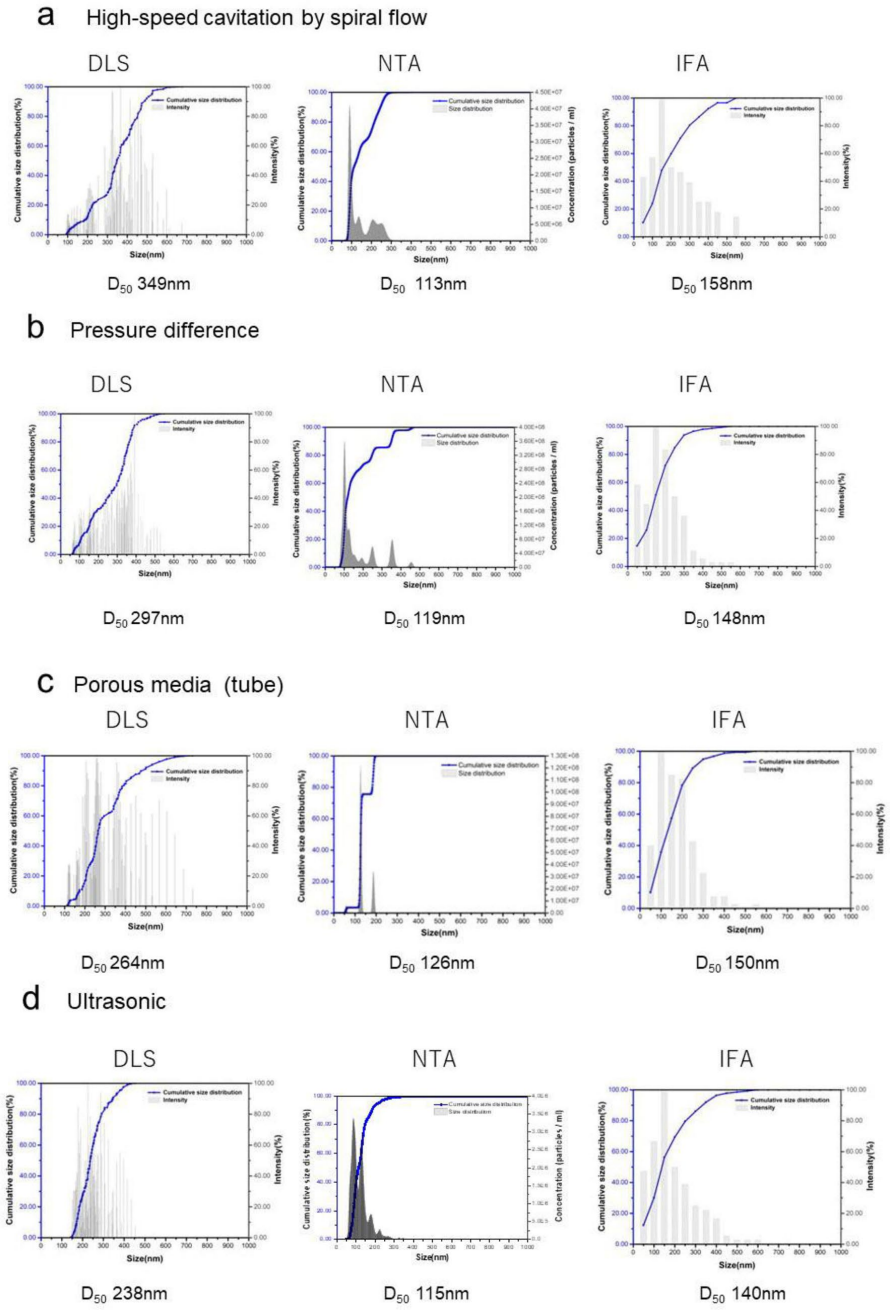
Nanobubble size distribution compared with different measurement methods at pH 10 water. (**a**) Nanobubble size distribution of three measurement methods (DLS, NTA, and IFA) and D_50_ produced by the spiral flow. (**b**) Nanobubble size distribution of three measurement methods (DLS, NTA, and IFA) and D_50_ produced by pressure difference. (**c**) Nanobubble size distribution of three measurement methods (DLS, NTA, and IFA) and D_50_ diameter produced by porous media (tube). (**d**) Nanobubble size distribution of three measurement methods (DLS, NTA, and IFA) and D_50_ produced by ultrasonication.

The size distribution of air nanobubbles prepared by four different methods were measured by DLS, NTA, and IFA; the results are shown in Fig. 3. The nanobubble size distribution producing different apparatuses was almost identical for each DLS, NTA, and IFA observation. The median diameter D_50_ in the nanobubble size distribution of NTA and IFA are similar at 150 nm. However, D_50_ in the size distribution of DLS is larger from 250 to 350 nm than those of NTA and IFA. Another report^52–54^ also showed that the observed DLS mean diameter was larger than NTA for nanobubbles, as DLS is more sensitive to large objects than small objects. Compared with solid less polydispersed particles such as gold (Fig. S5), the DLS of polydispersity of nanobubble size distribution could cause larger size distribution observations by the bias towards larger particles within the sample, as larger particles scatter light more intensely than smaller particles^53^. D_50_ of the nanobubble size distribution by different types of nanobubble production was 110–160 nm for NTA and IFA. However, the D_50_ of NTA is smaller than the D_50_ of IFA. To measure the particle diameter *d* in NTA^55^, the diffusion constant *D* is utilized by the Einstein-Stokes equation (Eq. 3).

3 $$D=\frac{kT}{3\pi \mu d}=\frac{{\overline{((x,y))}{}^{2}}}{4t}$$

where *k, T, µ, t* and ${\overline{(x,y)}}^{2}$ are the Boltzmann constant, absolute temperature, fluid viscosity, particle diameter, time, and mean square displacement, respectively, of the particle. Both NTA and DLS utilized the hydrodynamic diameter *d*. Although the nanobubble diameter estimates the bubble diameter and the thickness of the surface layer surrounding a bubble in the measurement by DLS^48^, the layer thickness surrounding a bubble is considered disregarded by NTA as NTA uses a sensitive camera and is analyzed by recorded videos^53^. Therefore, the nanobubble size measured by NTA is similar to that of the gas bubble size, not including the surface layer surrounding a bubble, similar to that measured by the solid gold particle size (Fig. S5).

On the other hand, IFA measured the physical diameter *d* + 2*δ* as a bubble diameter considering the surface layer surrounding a bubble, as shown in Fig. 2a2.

The measured size distribution of nanobubbles was not significant for the nanobubbles produced by porous media (tube) generation, as shown in Fig. 3c, as the bubbles were constantly produced by uniform porous size holes.

### Surface layer surrounding the gas nanobubble measured by IFA and NTA

**Figure 4:**
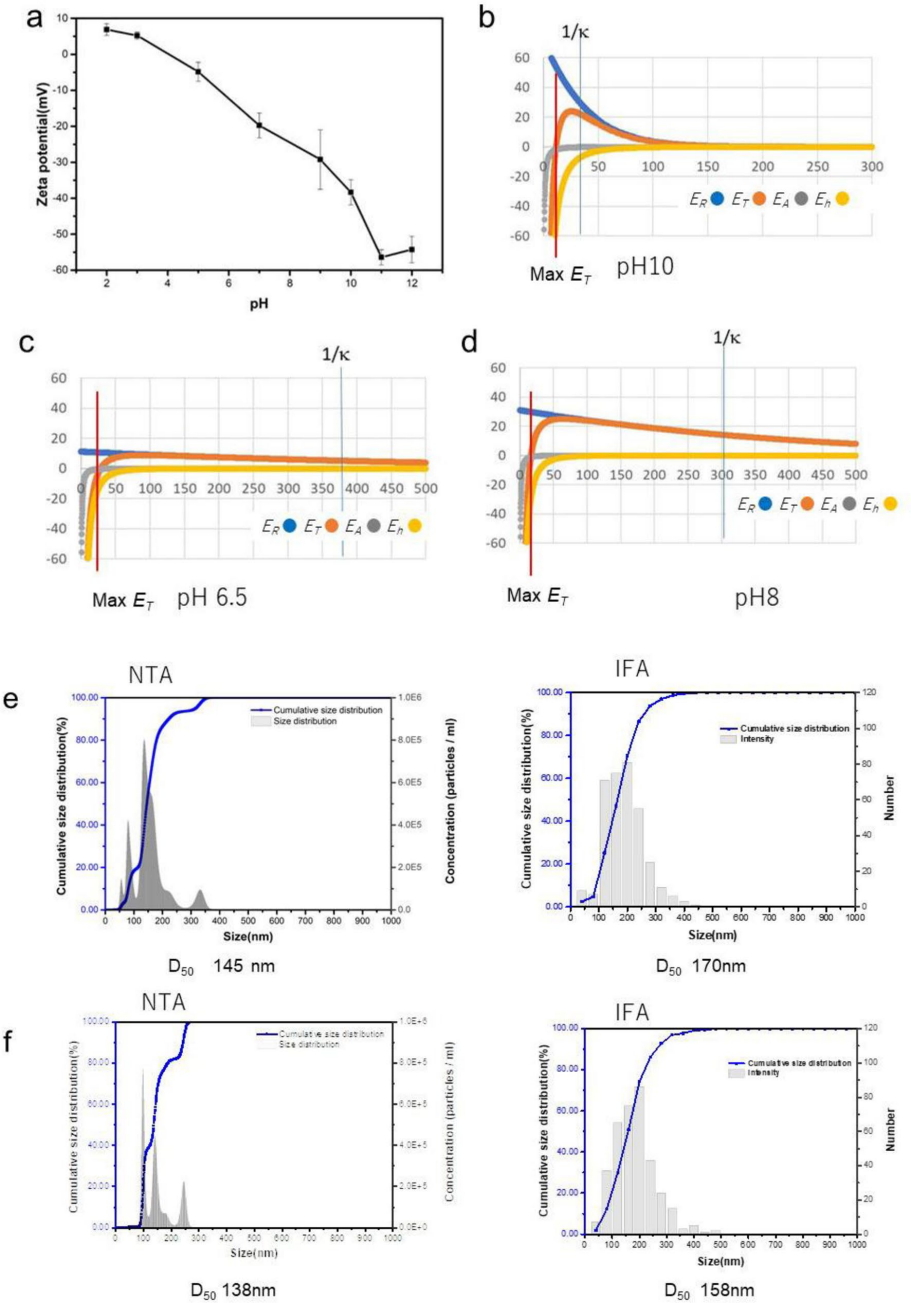
Nanobubble stability by two-particle interactions. (**a**) Zeta potential of air nanobubbles in water depending on pH. Air nanobubbles were produced by blowing air through porous media in deionized water. The total potential energy of nanobubbles ET, van der Waals energy EA, hydrophobic interaction energy Eh, and electrostatic interaction energy ER depending on pH are shown in b to d. The calculation method (refer to Supplementary Note 9). (**b**) Potential energies between two nanobubbles as a function of their separation distance at pH 10. (**c**) Potential energies between two nanobubbles as a function of their separation distance at pH 6.5. (**d**) Potential energies between two nanobubbles as a function of their separation distance at pH 8. (**e**) NTA and IFA nanobubble size distribution at pH 6.5 produced by porous media (tube). (**f**) NTA and IFA nanobubble size distribution at pH 8.0 produced by porous media (tube).

Nanobubble stability depending on solution pH, is summarized in Fig. 4. Many researchers^54–57^ have reported the stability of different nanobubbles in various conditions and concepts (Supplementary Table S2). The nanobubble surface in water is filled with different ions (H^+^, OH^−^, Na^+^, Cl^−^), which controls only pH to reduce the impurities and is estimated by extended Derjaguin–Landau–Verwey–Overbeek (DLVO) theory (refer to Supplementary Note 9, Fig. S6). The electrostatic repulsion of nanobubbles using DLVO theory is important to keep the stability of nanobubbles by causing the total potential energy barrier^46^. Using IFA measurement, evaluating a gas nanobubble’s actual physical diameter and the surface layer surrounding it is important. As shown in Fig. 4a, the air nanobubble isoelectric point is pH 3.8 in deionized water; therefore, here, the bubble size distribution was measured at an alkaline pH of 10, and the surface charge of the nanobubbles was higher negative than -50 mV, which supports that the nanobubbles were very stable, as explained by the extended DLVO theory. The interaction force of the two nanobubbles depended on their separation distance. It was expressed by the sum of the van der Waals interaction energy, hydrophobic interaction energy, and electrostatic interaction energy. In Fig. 4b–d, the electric double-layer thickness is denoted as 1/*κ*. As shown in the nanobubble size distribution at pH 10 in Fig. 3, the difference between D_50_ of IFA minus D_50_ of NTA was 30.8 ± 11.5 nm in a 95% confidence interval. Therefore, an approximately 10–20 nm thick surface layer surrounding the gas bubble existed when two nanobubbles contacted the direction of the electric field. This value was smaller than the electric double layer thickness of 30 nm at pH 10, as shown in Fig. 4b, and almost similar to the boundary of the positive total potential energy value distance, as the repulsion force between two nanobubbles could have appeared at a positive total potential distance. The calculated electric double layer thicknesses at pH 6.5 (Fig. 4c) and pH 8 (Fig. 4d) are much larger than 300 nm. By comparing NTA and IFA in Fig. 4e,f, the difference in D_50_ between NTA and IFA is 25 nm and 20 nm at pH 6.5 and 8.0, respectively. The surface layer surrounding the gas bubble comprised half of those differences. Therefore, approximately 13 nm at pH 6.5 and 10 nm at pH 8.0 estimated the boundary of the positive total potential energy value distance shown in Fig. 4c,d, respectively.

**Figure 5:**
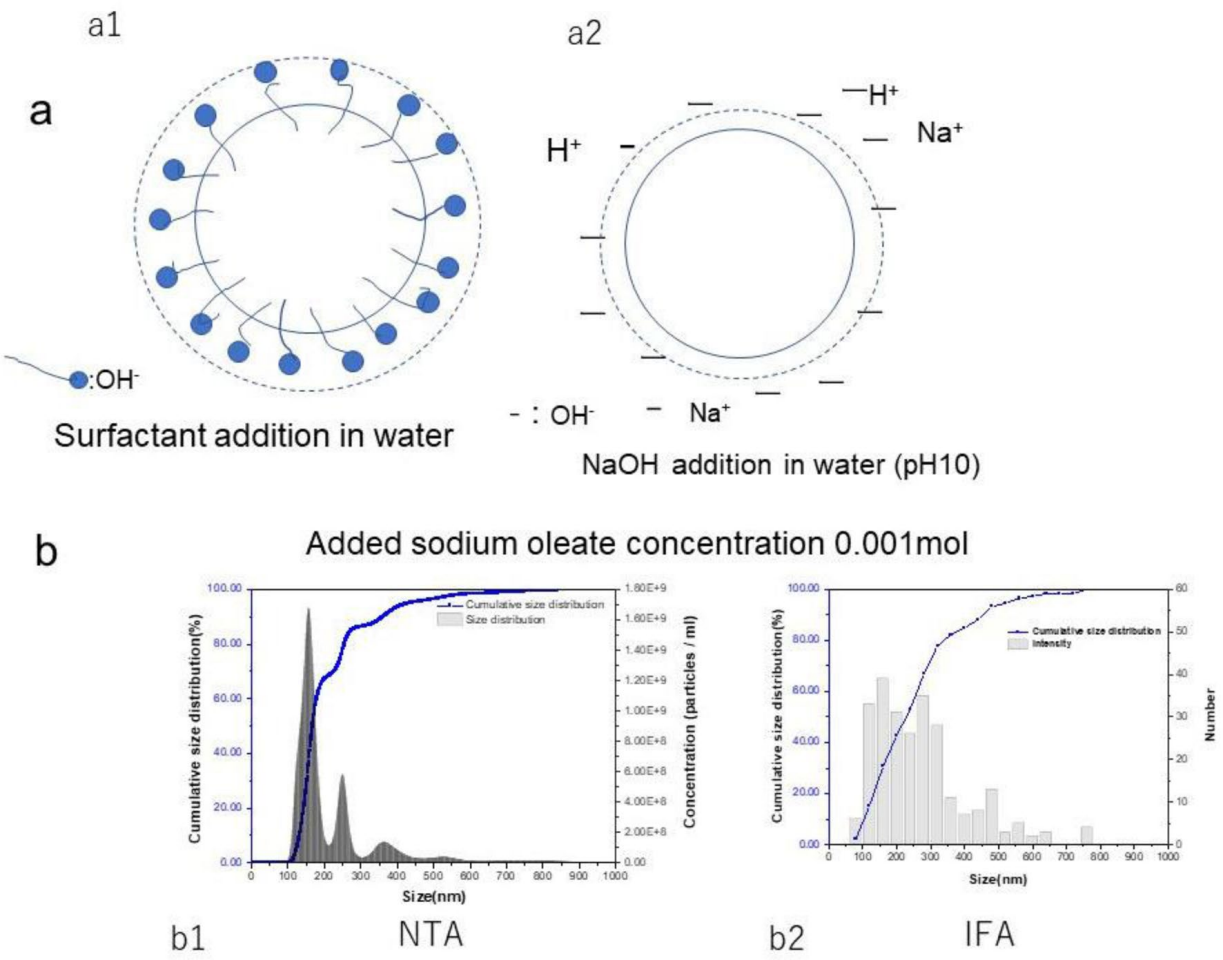
Size distribution of nanobubbles prepared in the presence of surfactant. (**a**) Model of surfactant adsorption onto a bubble surface in water at pH 10. (**a1**) Oleic ion-coated nanobubble. The apparent bubble size increased for the surfactant-coated layer at pH 10. Oleic acid was dissociated to 100% oleic ion. (**a2**) nanobubble in water at pH 10. No surfactant addition. (**b**) Size distribution by NTA and IFA. (**b1**) *D*_*50*_ is 160 nm; (**b2**) D_50_ is 181 nm. The surfactant thickness effect was assumed to be approximately 10 nm in 0.1 mmol/L sodium oleate.

Next, the nanobubble was prepared in an oleic ion surfactant-containing aqueous solution (0.1 mmol/L sodium oleate) as the length, and molecule surface area of the oleic ion were known^58,59^. The length of a surfactant molecule and the surface area of a bubble (or particle) occupied by a surfactant molecule’s tail (or head) is known, and the oleic ion is covered as a monolayer. Here, the effective covered oleic ion on the bubbles is calculated. Figure 5a shows the model of nanobubbles with surfactant (a1) and without surfactant (a2). The nanobubble concentration *C* (measured by NTA) was approximately 1 × 10^9^/mL, and the nanobubble size was *2a*. The total specific area *S*_*b*_ of bubbles per mL is calculated by the following equation:

4 $$S_{b} = C\pi a^{2}$$

when *2a* = 160 nm (Fig. 5b1), *S*_b_ = 1.5 × 10^−1^ cm^2^/mL. The nanobubble shows the bulk nanobubbles. Although there are size distributions on bubbles, the 50% average diameter is used in the calculation. Oleic ion covers the nanobubble as a monolayer, and the hydrophilic OH^−^ ions are arranged outside the bubble. All nanobubbles’ surface areas are estimated to be completely covered with many uniform oleic ion surface areas. It is calculated how much oleic concentration is necessary per unit volume. If one molecule’s surface area *S* of adsorbed oleic ion is estimated^60^ to be approximately 0.193 nm^2^, the oleic acid’s total surface area* S*_o_ per mL is shown in the following equation:

5 $$S_{o} = ASm$$

where *A* is the Avogadro number and *m* is the oleic ion concentration mol/L. When the oleic ion concentration was 0.1 mmol/L, the total surfactant area *S*_*o*_ per mL was 1.16 × 10^2^ cm^2^/mL.

When oleic ions are coated on all nanobubble surfaces, a large number of oleic ions remain in the aqueous solution. The difference between D_50_ 160 nm of NTA (Fig. 5b1) and *D*_*50*_ 181 nm of IFA (Fig. 5b2) was 21 nm. Therefore, the surfactant-covered layer was estimated to be approximately 10 nm, much larger than the oleic ion length^58^ of 2.59 nm. The surface layer surrounding the gas nanobubbles on the surfactant-coated nanobubbles was similar to the surface layer with no oleic acid addition (*D*_*50*_ of IFA minus *D*_*50*_ of NTA), as shown in Fig. 3a–d.

Here, the nanobubble stability is examined in kerosene as the organic solvent. The small amount of moisture shows the zeta potential of nanobubbles in kerosene measured by the DLS systems. The nanobubbles in kerosene are not stable as nanobubbles in water.

IFA and NTA were 161 and 147 nm on the day they were prepared, the first day, and decreased to 116 and 108 nm, respectively, after 10 days (Supplementary Note 9. Nanobubble size distribution in kerosene by IFA and NTA, Fig. S6). The covered layer of nanobubbles was estimated to be 7 nm on the first day and decreased to 4 nm after 10 days. If the pressure by the Young–Laplace equation is equal to that by the electrostatic force, the nanobubble diameter *d* is expressed as shown in the next equation^53^:

6 $$d = \frac{2\gamma \epsilon }{{\pi \sigma^{2} }}$$

where *g, s,* and *e* are the liquid’s surface tension, surface charge density, and permittivity, respectively. The *g* of kerosene with air is 26 × 10^−3^ N/m, 2.8 times smaller than the *g* of water (72.7 × 10^−3^ N/m)^61^. On the other hand, the zeta potential of nanobubbles in kerosene is 10 times smaller, that is, − 3.9 ± 1.4 mV (Supplementary Fig. S7), than that in deionized water, which is − 20 to − 40 mV (Fig. 4a). The permittivity^62^ ratio of kerosene per water is approximately 0.03. Therefore, *d* is proportional to 0.36 × 0.03/(0.1)^2^ in Eq. (6). The diameter of nanobubbles in kerosene is slightly larger than that of nanobubbles in deionized water. As shown in Supplementary Fig. S7, D_50_ of the nanobubbles in kerosene was 161 nm and 147 nm by IFA and NTA, respectively, on the first day and was slightly larger than *D*_*50*_ for deionized water at pH 10 (Fig. 4). However, the zeta potential of nanobubbles in kerosene was very small (− 3.9 ± 1.4 mV); therefore, the nanobubbles in kerosene were not stable. After 10 days, the nanobubble concentration decreased to 3.5% compared with the initial bubble concentration, and the *D*_*50*_ of the nanobubbles also decreased.

## Conclusions

Our findings established a methodology by using novel equipment (interactive force, IFA) to measure the physical nanobubble size distribution of gas bubbles and the surface layer by measuring the attractive and repulsive forces of physical forces caused by a small distance in nm change between hemispherical electrodes and flat electrodes in nanobubbles containing liquid under an electric field. Additionally, the surface layer surrounding gas nanobubbles in different conditions of the liquid was measured for the first time, and unique and important nanobubble characteristics were measured by IFA. This equipment can also be applied in complex systems, especially in liquids with poor light transmittance, high bubble concentration, and lower reflective index between bubble and liquid.

## Supplementary Information

Supplementary Information.

## Data Availability

Data supporting this study’s findings are available from the first author and corresponding author upon reasonable request. In addition, some measured raw data are shown in Excel files. Model Description: All of the image processing in this work was performed using the MATLAB Image Processing Toolbox, which provides an extensive set of reference-standard algorithms and functions for image processing, analysis, visualization, and algorithm development. The weight data read by the balance are applied to derive the displacement and to obtain a set of undulating curves. First, the MATLAB software smoothed the curve, named medfilt1, to median filter the noisy sine wave signal y and draw the waveform. Second, we specify a window width of 5 and perform median filtering on y. Last, we identify the peak value and determine that the interval between the two peaks represents particle size information. This process was repeated to obtain all statistical information.
