# Supplementary material for: Nanobubble size distribution measurement by interactive force apparatus under an electric field
Source: Sci Rep. 2023 Mar 4;13:3663. doi: 10.1038/s41598-023-30811-9 (PMC9985613; doi:10.1038/s41598-023-30811-9)
Supplement: Supplementary file 1 — Supplementary Information. [file 41598_2023_30811_MOESM1_ESM.docx]

Supplementary Information:

**Nanobubble size distribution measurement by interactive force apparatus under an electric field**

**Zhenyao Han^1^, Chen Hao^1^, He Chunlin^1^, Gjergj Dodbiba^2^, Akira Otsuki^3,4^, Yuezhou Wei^5^, Toyohisa Fujita^1^***

1. School of Chemistry and Chemical Engineering and College of Resources, Environment and

Materials, Guangxi University, Nanning 530004, China; 1622303001@st.gxu.edu.cn (Z.H.);

hchen1996@st.gxu.edu.cn(H.C.) helink1900@126.com (C.H.); fujitatoyohisa@gxu.edu.cn (T. F.)

2. Graduate School of Engineering, The University of Tokyo, Bunkyo 113-8656, Japan; dodbiba@g.ecc.u-tokyo.ac.jp (G.D.)

3. Facultad de Ingeniería y Ciencias, Universidad Adolfo Ibáñez, Diagonal Las Torres 2640, 11 Peñalolén, Santiago 7941169, Chile, akira.otsuki@uai.cl (A.O.)

4. Waste Science & Technology, Luleå University of Technology, SE 971 87 Luleå, Sweden (A.O.)

5. School of Nuclear Science and Technology, University of South China, Hengyang City, Hunan, 421001, China; yzwei@usc.edu.cn (Y.W)

* Corresponding author (T.F.)

This file includes the following:

Supplementary Notes 1 to 10

Figure S1 to S7

Table S1 to S2

**S****upplementary Note 1. Particle size range to measure**


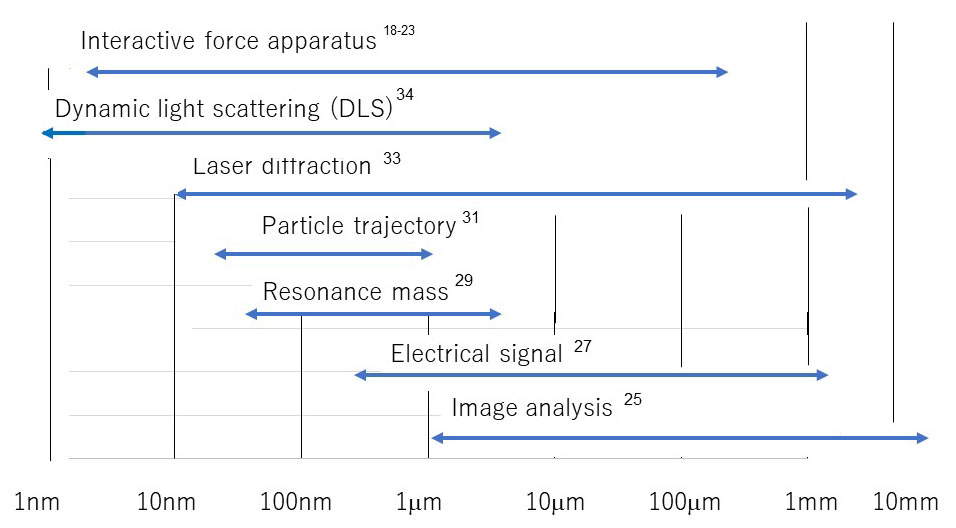


**Figure S1.** Particle size range can be measured by different particle size measurement methods.

**Supplementary Note 2. Particle size measured by interactive force apparatus**

**Table S1. Particle size (mean diameter) measured by IFA**

**
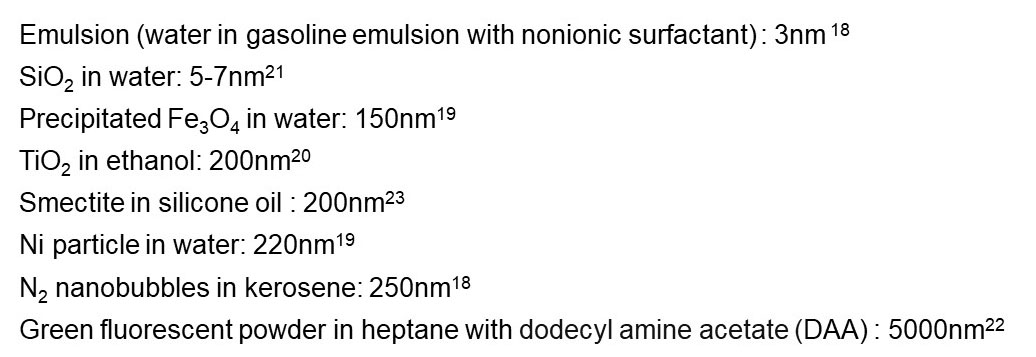
**

**Supplementary Note 3. Concept of surface layer surrounding gas nanobubble**

**Table S2. Various concepts of surface layer surrounding gas nanobubble to stabilize**

| ・Electrical double-layer [1-3]  Hydroxyl ions [4]  Electrical double-layer + hydrogen bond [5]  ・Hard hydrogen bonds [6-8]  Super solid skin [9]  ・Contaminants [10]  ・Organic skin or film with surfactant [11,12]  Hydrophobic material [13]  Protein [14] |
| --- |

**Supplementary Note 4. Nanobubble preparation equipment**


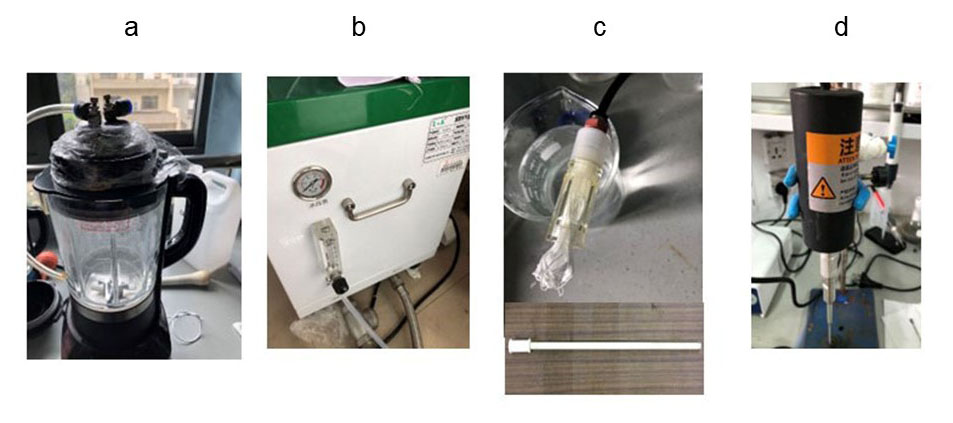


**Figure S2.** Four types of nanobubble preparation methods in this experiment.

**a**, High-speed cavitation equipment (homemade equipment) by the spiral flow. A total of 750 mL of deionized water, adjusted to pH 10 or natural pH, was fed to the vessel to immerse the blade. The rotation speed was 18000 rpm, i.e., the peripheral velocity was 67.8 m/s. The total agitation time was 10 minutes, and the discontinuous stirring method was adopted. Stirring was paused every minute to avoid overheating. **b**, The ultra-nanobubble generator (XZCP-K-1.1) produced by Xiazhichun Co. Ltd. (Kunming, China) by pressure difference. Five liters of deionized water were adjusted to pH 10 and allowed to move for 15 minutes. The air intake was 2 Nl/min, and the water pressure in the tank was controlled within 0.40-0.45 MPa. **c**, Ultrafine bubble (UFB) generator (KITZ MICRO FILTER CORPORATION, Japan) by porous media (tube). The air flowed at 100 mL/min for 3 hours to prepare nanobubbles in 200 ml of deionized water. The pore size of the tube is approximately 1~2 μm, as observed by SEM. **d**, Ultrasonic generator hup-100, produced by Tianjin Heng/ao Technology Development Co., Ltd. (Tianjin, China). The preparation time was 10 min in total in 100 mL, and the intermittent working mode was adopted. Each time the ultrasonic wave is used for 30 s, the interval is 30 s. According to the equipment model, the power of this instrument is 80 W, and the frequency is 20 kHz.

**Supplementary Note 5. Force between two particles by applying an electric field**

As shown in Figure 2b, the force between two spherical particles under an electric field is considered. When the two spherical particles are contacted, the force to the y-axis *F_y_* and the force to the x-axis *F_x_* are shown in the following formula.

$F_{y}=-\frac{1}{4\pi\varepsilon_{0}\varepsilon_{r}}\frac{3{(PV)}^{2}}{r^{4}}\times\left[ \left( 3{cos\theta}^{2}-1)sin\theta+cos\theta sin2\theta\right. \right]$ (1)

$F_{x}=-\frac{1}{4\pi\varepsilon_{0}\varepsilon_{r}}\frac{3\left( PV \right)^{2}}{r^{4}}\times\left[ -(3{cos\theta}^{2}-1)cos\theta+sin\theta sin2\theta\right]$ (2)

where *ε*_o_ is space permeability, *ε_r_* is relative permittivity, and *r* is the distance between the centers of two spherical particles.

Here, $\varepsilon_{0}$=8.854x10^-12^(F/m), $\varepsilon_{r}=80(-),$*P* =$\varepsilon_{0}\varepsilon_{r}$*E, E=*1/r(V/m), r= 1～ 1000 (nm),

V = (4/3) π(r/2)^3^, angle $\theta$is 0 to π/2 were used in the experiment.

**Supplementary Note 6. Simulation of forces between two electrodes dispersing various size distributions of particles by changing the electrode distance**


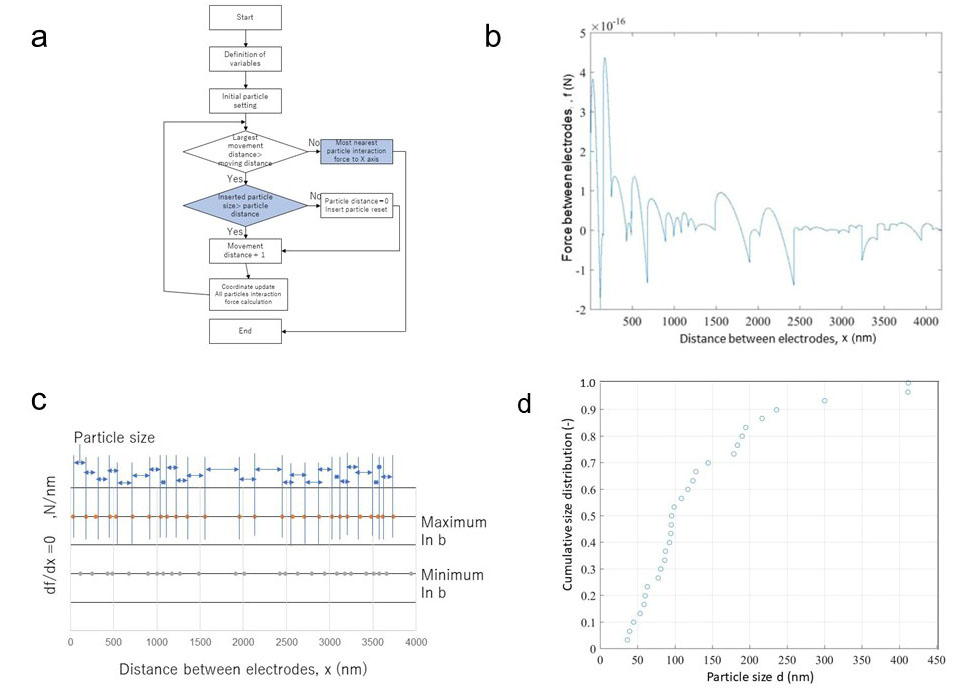


**Figure S3. a,** Algorithm to calculate forces between two electrodes dispersing various size distributions of the particle by changing the electrode distance. **b**, One example of calculated force is when various size of particles is dispersed. The force between electrodes by changing the electrode distance dispersing the size of particles by the calculation using equations (2) and (3). Logarithmic normal random average 50 nm, Distance to measure 3000 nm, Logarithmic normal random variance 1000 nm^2^. **c**, Differentiation of force versus distance and d*f*/d*x* = 0 on maximum and minimum in b are shown. The peak-to-peak shows particle size, and the different particle sizes can be accumulated. **d**, Simulated cumulative size distribution curve by using c) data. The median diameter (50% of particle diameter in the cumulative size distribution) is around 100 nm in this condition.

**Supplementary Note 7.** **Voltage effect of nanobubble size distribution by IF**


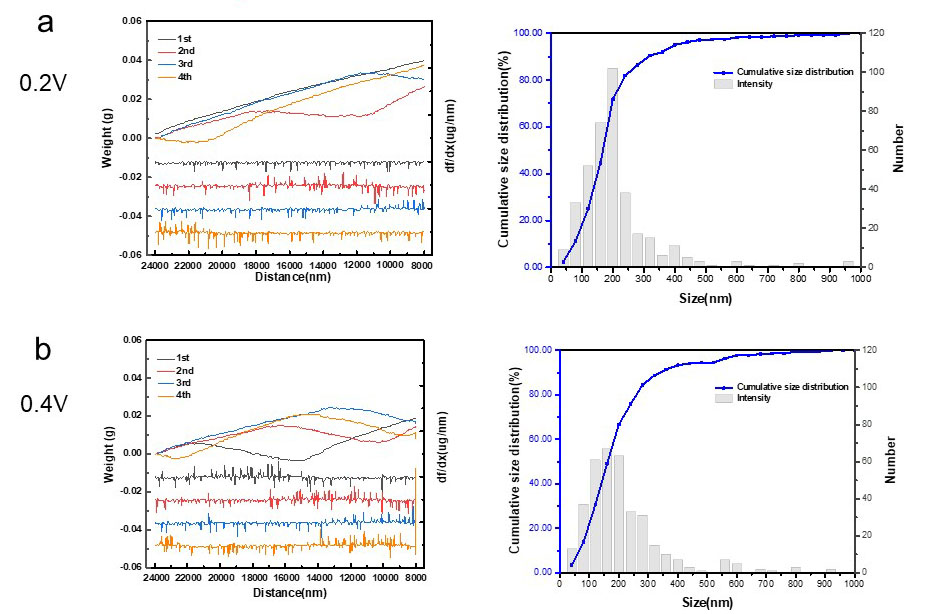


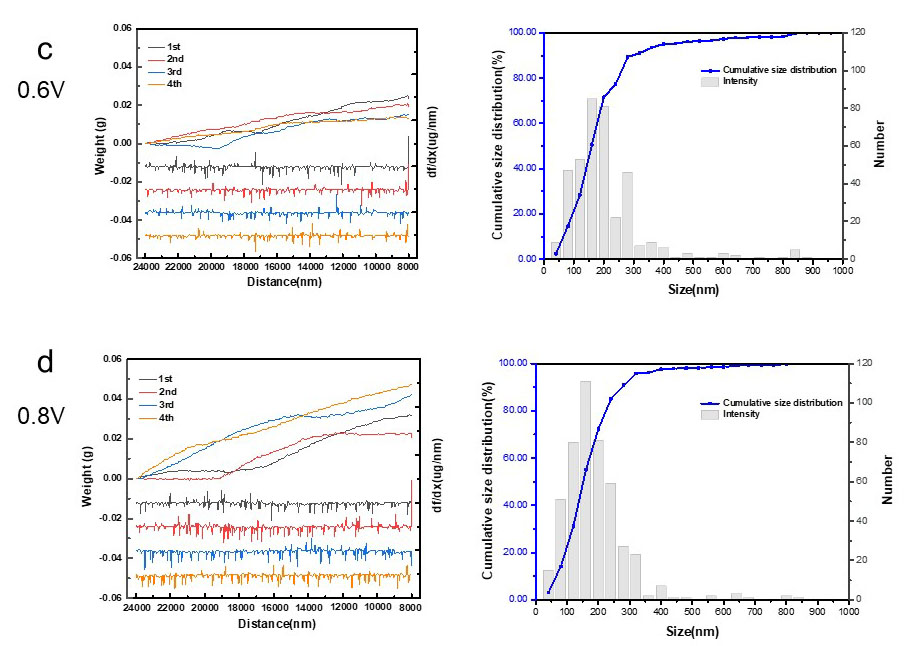


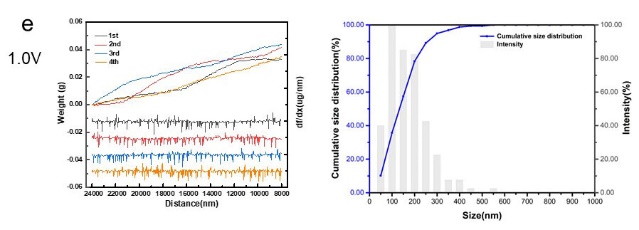


**Figure S4.** The voltage effect of measured forces by approaching the electrodes, as shown in Figure 2a, and the nanobubble size distributions by IFA. Air nanobubble was produced by the method of Figure 7 at pH 10. **a**, 0.2V, D_50_ 141 nm, **b**, 0.4V, D_50_ 137 nm, **c**, 0.6V, D_50_ 150 nm, **d**, 0.8V D_50_ 137 nm, **e**, 1.0V D_50_ 136 nm. (Here, 50% diameter “D_50_” in cumulative size distribution was used as the median diameter. On the other hand, particle number in size area x size /total particle number was used as mean diameter.)

**Supplementary Note 8.** **Solid gold nanoparticle size distributions compared DLS, NTA, IFA, and SEM.**


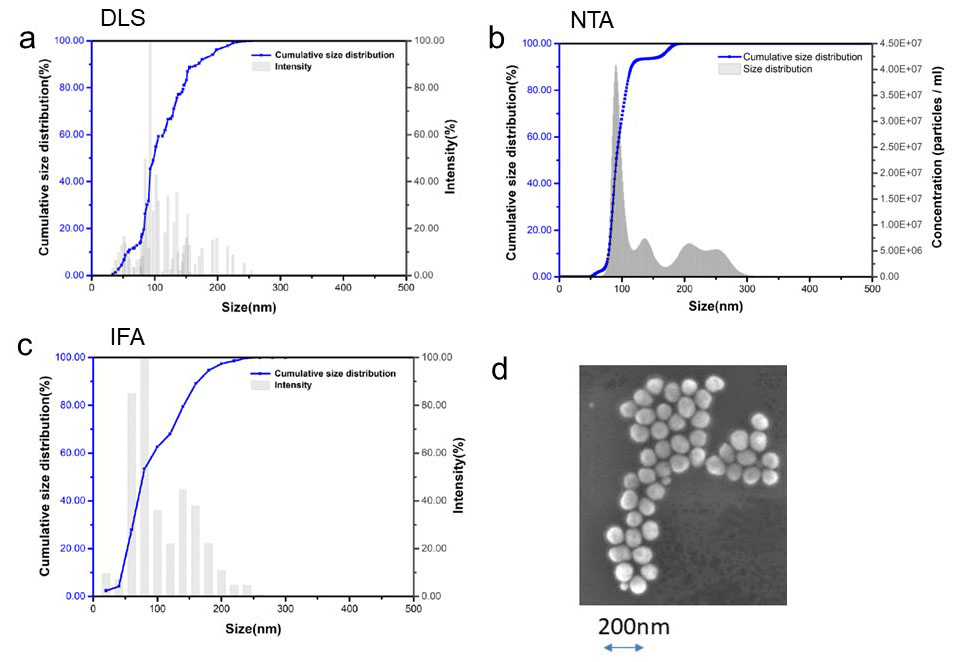


**Figure S5.** Solid gold nanoparticle size distributions were comparing DLS, NTA, and IFA. The gold particles were dispersed in water. a, Size distribution measured by DLS. D_50_ 95 nm. b, Size distribution measured by NTA. D_50_ 92 nm c, Size distribution measured by IFA. D_50_ 90 nm d, D_50_ 98 nm by SEM image analysis.

**Supplementary Note 9. Interaction force between two nanobubbles in water**

***
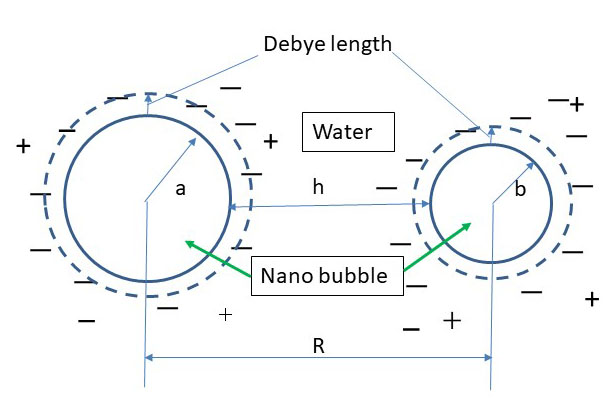
***

**Figure S6**. Position of two kinds of nanobubbles.

If two nanobubbles exist in water, their position can be defined as shown in Supplementary Figure 5.

$R=a+b+h$ (3)

The total potential energy *E_T_* between two nanobubbles can be the sum of van der Waals interaction energy *E*_A_, hydrophobic interaction energy *E_h_*, and electrostatic interaction energy *E_R_*.

$E_{T}=E_{A}+E_{h}+E_{R}$ (4)

These potential energies can be compared with the *kT* unit composed of the Boltzmann constant *k* and absolute temperature *T.*

$E_{T}/kT={(E}_{A}+E_{h}+E_{R})/kT$ (5)

The sum of van der Waals interaction (*E_A_*) and hydrophobic interaction (*Eh*) is shown in the following formula.

$E_{A}+E_{h}=-\frac{\left( A+K \right)}{6}\left[ \frac{2ab}{R^{2}-{(a+b)}^{2}}+\frac{2ab}{R^{2}-{(a-b)}^{2}}+ \ln\left( \frac{R^{2}-\left( a+b \right)^{2}}{R^{2}-\left( a-b \right)^{2}} \right) \right]$ (6)

where *A* is the Hamaker constant, and *K* is the hydrophobic constant.

The Hamaker constant A for air in water is used 3.7x10^-20^ J [15], and the *K* is a hydrophobic constant for air in the water and 10^-18^ J in deionized water [16].

If $\psi_{1}$and$\psi_{2}$are surface potentials of nanobubbles radii a and b, respectively, the electrostatic potential energy (*E_R_*) is shown in the next formula.

$E_{R}=-\frac{\pi\varepsilon_{r}\varepsilon_{0}ab\left( \psi_{1}^{2}+\psi_{2}^{2} \right)}{a+b}\left[ \frac{2\psi_{1}\psi_{2}}{\psi_{1}^{2}+\psi_{2}^{2}}ln\frac{1+\exp\left( -\kappa h \right)}{1-\exp\left( -\kappa h \right)}+\ln[1-\exp\left( -2\kappa h \right) \right]$ (7)

where $\varepsilon_{r}$ represents the relative dielectric constant, $\varepsilon_{0}$ represents the permittivity of the vacuum.

The thickness of the electric double layer can be represented by the Debye length ($\lambda_{D}=1/\kappa$), which can be described by the following formula:

$\kappa=\sqrt{2nz^{2}e^{2}/{(\varepsilon}_{r}\varepsilon_{0}kT)}$ (8)

$n=1000N_{A}C$ (9)

where $n$ is the number concentration of anion or cation in the solution, $z$ represents the valence of the ion, $e$ is the electron charge, $N_{A}$is the Avogadro number and $C$ is the concentration of anion or cation (mol/L)

**Supplementary Note 10.** **Nanobubble size distribution in kerosene by IFA and NTA**


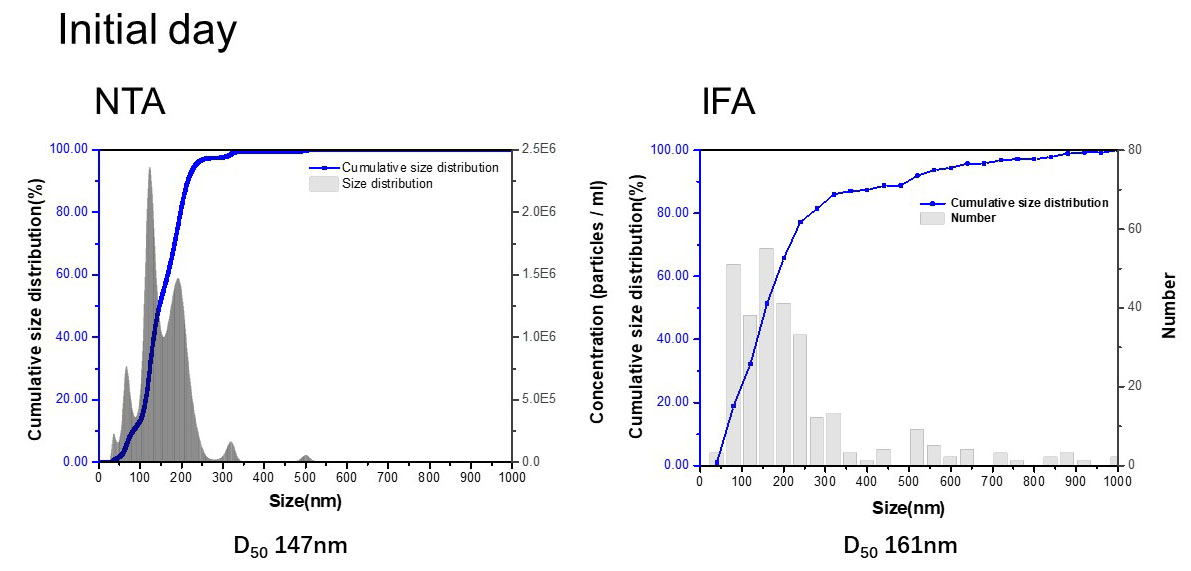


**b**


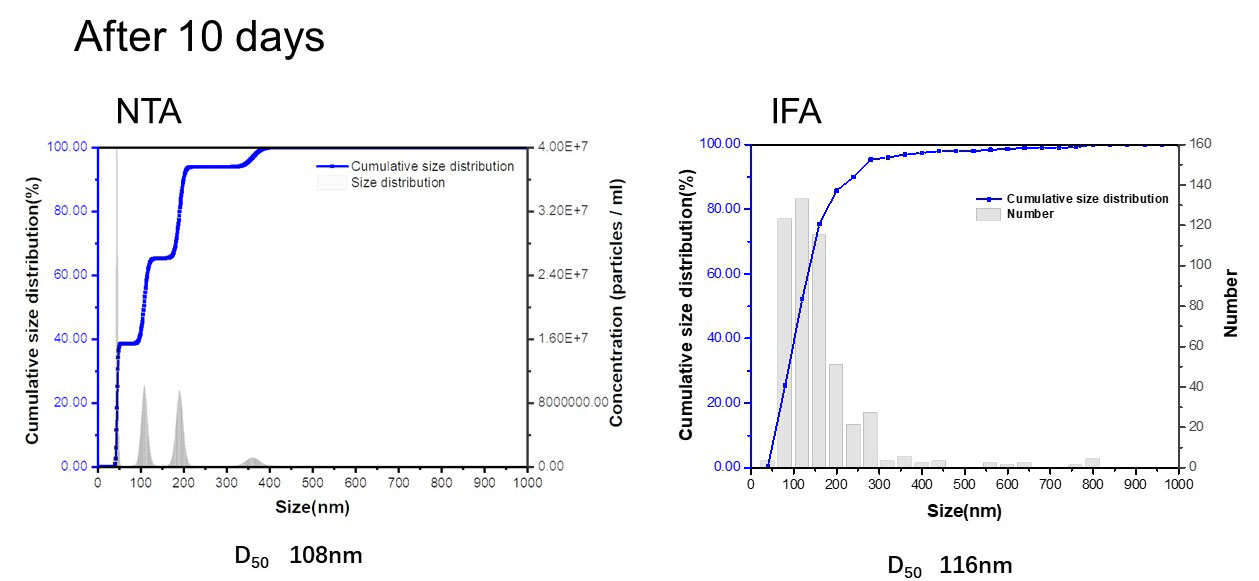


**Figure S7**. Nanobubble size distribution in kerosene was measured by NTA and IFA prepared by porous media (tube) for 3 hours on the initial day and after 10 days. Zeta potential of nanobubbles in kerosene: -3.9±1.4 mV (measured by DLS system.) a) D_50_ of IFA-D_50_ of NTA =14 nm, therefore the covered layer of nanobubble in kerosene can be 7 nm. The bubble concentration measured by NTA was 2.07x10^8^ bubble /cm^3^. b) D_50_ of IFA-D_50_ of NTA =8 nm. Therefore, the covered layer of nanobubble in kerosene can be 4 nm. The bubble concentration measured by NTA is 7.18x10^6^ bubble /cm^3^.

**Supplementary References**

1. Hewage, S. A., Kewalramani, J. & Meegoda, J. N. Stability of nanobubbles in different salts solutions. Colloids and Surfaces A: Physicochemical and Engineering Aspects 609, 125669 (2021). doi:https://doi.org/10.1016/j.colsurfa.2020.125669

2. Meegoda, J. N., Hewage, S.A. & Batagoda, J.H. Application of the Diffused Double Layer Theory to Nanobubbles, Langmuir, 35, 121000-12112 (2019)

DOI: 10.1021/acs.langmuir.9b01443

3. Ma, J. et al., Ion adsorption stabilizes bulk nanobubbles, J Colloid Interface Sci, 606, 1380-1394 (2022) https://doi.org/10.1016/j.jcis.2021.08.101

4. Satpute, P. A. & Earthman, J. C. Hydroxyl ion stabilization of bulk nanobubbles resulting from microbubble shrinkage. Journal of Colloid and Interface Science 584, 449-455 (2021). doi:https://doi.org/10.1016/j.jcis.2020.09.100

5. Bu, X. & Alheshibri, M. The effect of ultrasound on bulk and surface nanobubbles: A review of the current status. Ultrasonics Sonochemistry 76, 105629 (2021). doi:https://doi.org/10.1016/j.ultsonch.2021.105629

6. Ohgaki, K., Khanh, N. Q., Joden, Y., Tsuji, A. & Nakagawa, T. Physicochemical approach to nanobubble solutions. Chemical Engineering Science 65, 1296-1300 (2010). doi:https://doi.org/10.1016/j.ces.2009.10.003

7. Michailidi, E. D. et al. Bulk nanobubbles: Production and investigation of their formation/stability mechanism, J Colloid Interface Sci, 564, 371-380 (2022)

8. Hong, S.-N., Ri, J.-H., Mun, S.-Y. & Yu, C.-J. Revealing the influence of porosity and temperature on transport properties of nanobubble solution with molecular dynamics simulations. Journal of Molecular Liquids 367, 120518 (2022). doi:https://doi.org/10.1016/j.molliq.2022.120518

9. Zhang, Xi et al., Nanobubble Skin Supersolidity, Langmuir, 32, 11321-11327 (2016)

10. Ducker, W.A., Contact angle and stability of interfacial nanobubbles. Langmuir, 2009. 25(16): p. 8907-10. DOI: 10.1021/la902011v.

11. Fox, F. E. & Herzfeld, K. F. Gas Bubbles with Organic Skin as Cavitation Nuclei. The Journal of the Acoustical Society of America 26, 984-989 (1954). doi:10.1121/1.1907466

12. Yoon, R.H. & Askoy, B.S. Hydrophobic forces in thin water films stabilized by dodecyl ammonium chloride, J Colloid Interface Sci,211 1-10 (1999)

13. Yasui, K., Tuziuti, T. & Kanematsu, W. Mysteries of bulk nanobubbles (ultrafine bubbles); stability and radical formation, Ultrason Sonochem, 48 259-266 (2018).

14. Hirai, M. et al., Structure of ultrafine bubbles and their effects on protein and lipid membrane structures studied by small- and wide-angle X-ray scattering, J Phy. Chem B 123 3421-3429 (2019).

15. Israelachivili, J. N. Intermolecular and surface forces with Applications to Colloidal and Biological Systems. Academic Press Limited, London, UK (1995)

16. Wang. L. & Yoon, R.H. Hydrophobic forces in the foam films stabilized by sodium dodecyl sulfate; Effect of electrolyte, Langmuir, 20, 11457 (2004)
